# Principal Approaches to Understanding Occupation and Occupational Science Found in the Chilean Journal of Occupational Therapy (2001–2012)

**DOI:** 10.1155/2017/5413628

**Published:** 2017-05-21

**Authors:** Rodolfo Morrison, Silvia Gómez, Enrique Henny, María Jesús Tapia, Laura Rueda

**Affiliations:** ^1^Department of Occupational Therapy and Occupational Science, Faculty of Medicine, University of Chile, Santiago, Chile; ^2^Chilean Society of Occupational Science, Santiago, Chile; ^3^Tedes Center, Santiago, Chile

## Abstract

**Background:**

The progression of occupational science in Chile is documented in the main scientific publication of the field, the Chilean Journal of Occupational Therapy (RChTO).

**Objective:**

Identify approaches to understanding and applying occupation and occupational science as elucidated in the RChTO.

**Methodology:**

A systematic qualitative review of the journal (2001–2012) identified articles elucidating an approach to understanding and application operationally defined as references to specific authors, theories, models/paradigms, definitions, and other fields that support approaches to O/OS.

**Results:**

The study identified two main approaches. The first considers occupation/occupational science from a practical perspective or as a means to explain human behavior; the second considers occupation/occupational science as an object of study. Each approach is further divided into categories.

**Conclusion:**

This study provides a novel perspective on regional use of occupational science concepts. These findings contribute to our understanding of this science in context and to recognition of the cultural relevance of these scientific concepts.

## 1. Introduction

Rapid technological advances and the emergence of the information society have changed the landscape for the scientific community. It is more important than ever to elucidate the international perspectives and bodies of knowledge that form the foundation of any discipline. Given the abundance of information available, it is essential to provide clear guidelines for discourse and academic research.

Occupational therapy in Chile has been influenced by occupational science concepts that were developed in other regions but are applicable to the Chilean context. However, undergraduate occupational therapy programs tend to teach a limited number of theoretical frameworks [1]. Moreover, within our country there has been little discussion of the various ways that occupational science and occupation can be understood and the local implications of these alternative perspectives [2–4].

The challenge for Chilean occupational therapists is to stimulate local discussion of these ideas and develop a theoretical framework that will support undergraduate and postgraduate occupational science/occupational therapy education. This content would provide students with a sound theoretical foundation, strengthen professional identity, and create effective connections between theory and practice [5–11].

Meeting this challenge requires recognizing the place of occupational science in professional education. Studies have shown that occupational therapists rarely use occupational therapy-specific theories to justify their practices, depending instead on concepts borrowed from other disciplines. This situation can be improved if occupational science concepts, foundations, and theories are taught explicitly [12] in occupational therapy education programs, along with their therapeutic applications to occupational behaviors.

As members of the Occupational Science Education Committee, we propose that it is essential to analyze the Chilean understanding of occupational science. Chilean perspectives are reflected in the Chilean Journal of Occupational Therapy (RChTO), which is our highest-impact scientific journal in the discipline [13]. This study explores the question: What approaches to understanding and applying occupational science/occupation are referenced in RChTO articles?

*Theoretical Framework*. As the objective of this study is to identify approaches to understanding and applying occupational science, we will define some relevant concepts.

Occupational science is the academic study of occupation [14]. It provides a theoretical and scientific foundation for occupational therapy practice, approaching occupation as an inherent human phenomenon. Because of its focus on human behavior, occupational science is considered a social science, like anthropology, psychology, or sociology [15].

One of the first approaches used in occupational science was to study the form, function, and meaning of occupation [14]. Form references to directly observable aspects of occupation. For example, occupational scientists may compare how different cultures or social groups perform a given occupation. Function refers to the contributions of an occupation to human development, health, or quality of life. Meaning refers to the subjective experience of participating in an occupation, which is constructed symbolically within a culture.

Understanding the evolution of occupational science/occupational therapy requires epistemological reflection. Contemporary analysis also involves social, economic, and political dimensions, as the discipline has broadened from an individual-centered perspective to consider the contribution of the community and context [16].

Occupational science/occupation is addressed in many ways in the literature. One way to sift through these perspectives is to investigate how concepts are applied, understood, and studied within the field, seeking to identify different approaches to understanding and application.

Several authors [17–19], reflecting on currents of thought, define approaches to understanding and application as ideologies, viewpoints, or theoretical concepts which explain phenomena that are shared, followed, and reproduced [18] within a discipline [19] through conferences, scientific journals, or other means. Authors sharing an approach may develop their own branches of the fundamental theory but use a common explanatory model [17]. Therefore, an approach is distinctive within a discipline [20], and there is general agreement on the basic conceptual elements and theoretical foundations [18].

Occupational therapy paradigms represent unified concepts set forth by authors seeking to define the nature and purpose of the discipline. These concepts are based on the principles articulated in publications such as those listed in the bibliography. Paradigms provide conceptualizations that organize theories and explain their applications [20].

Approaches to understanding and application represent a distinctive vision of the world. Different approaches may share instruments or theoretical categories. The procedures used in different approaches are based on their underlying world view [18]. Authors sometimes explicitly identify an approach as separate from another approach to understanding and application, alluding to the controversies over different approaches [19].

## 2. Methods

A systematic qualitative review [21] of the RChTO was performed. This method is based on grounded theory [22], which identifies the core concepts within systematically collected data on a topic. Such an approach does not require an a priori hypothesis but rather seeks to identify a new theory, in this case, approaches to the study of occupation. Other reviews have found this method effective for discovering patterns in the occupational science literature [23].

Inclusion criteria for articles were as follows: (a) keyword(s) and/or title referring to OS/O concepts; (b) published articles between 2001 and 2012; and (c) being available in full-text format.

Articles were analyzed using the operational definition of approach to understanding and application:

- *Authors refer explicitly* to a viewpoint on occupational science/occupation.
- *Theories are explicitly identified*, such as epistemological assumptions in the context of a given paradigm; general theories on society or history; theories or assumptions on measurement, observation, data collection, or empirical evidence; theoretically based inferences; or construction of theoretical concepts [24].
- *Conceptual models and paradigms are used*. For instance, theoretical or conceptual elements extracted from a general theory are cited; a model is used to understand an issue; or an explicit theoretical position from a specific approach is applied.
- *Definitions of occupational science/occupation are provided*. A definition is an attempt to explain an idea or term by providing a precise and exhaustive reflection. Therefore, a definition is a cohesive description of the complex state of an object, circumstance, or idea that can be validated through examination.
- *Conceptualizations of occupational science/occupation are developed*. A conceptualization is an abstract, simplified perspective on our understanding of some aspect of the world. These abstract ideas stem from the experience and conscious understanding of the world [24, 25]. Conceptualizations may include new propositions and concepts or syntheses of existing ideas on occupational science/occupation.

The research team held several meetings to agree on our understanding and synthesis of the content and capture the issues highlighted by the authors. Finally, the articles were categorized according to similarities and differences.

The research team systematically carried out specific tasks, including cross-reviewing notes, working in parallel and in dyads, outlining meeting minutes, summarizing findings, and critically reviewing these summaries.

The process can be summarized as follows:

- Article selection.
- Identification and labeling of references to occupational science/occupation, including authors referenced, in each full-text article.
- Summaries of findings regarding the approaches cited, including the key ideas and citations to support each point.
- Triangulation of information gathered by the research team.
- Analysis and synthesis of articles in weekly meetings.
- Categorization of articles.
- Naming the approach to understanding and application for each group.

## 3. Analysis and Results

**Figure 1:**
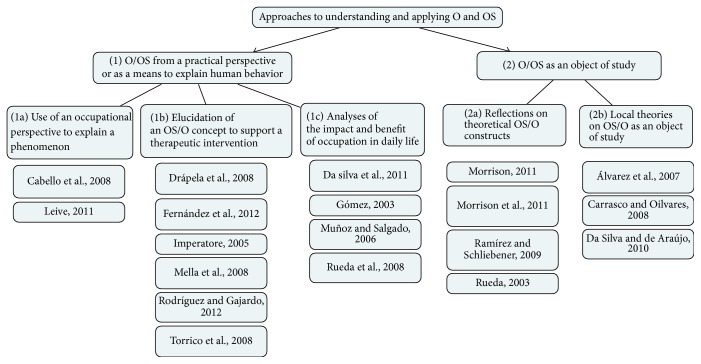
Approaches used by various authors, highlighting the two main approaches and the categories.

Of the 95 articles published in the journal between 2001 and 2012, 19 met the inclusion criteria. The analysis identified two main approaches. The first considers occupation/occupational science from a practical perspective or as a means to explain human behavior (12 articles); the second considers occupation/occupational science as an object of study (7 articles). Both approaches were then divided into categories, as shown in Figure 1 and described below.

*(1) Occupation or Occupational Science Considered from a Practical Perspective or as a Means to Explain Human Behavior*. These approaches represent first-order analyses, that is, studies performed by authors who instead of asking “what is occupation?” attempt to assign attributes to the concept and provide bibliographic references to support their analysis. These authors approach occupational science/occupation from a practical standpoint and as a way to explain human behavior.

This approach is divided into categories: (a) use of an occupational perspective to explain a phenomenon; (b) elucidation of an occupational science/occupation concept to support a therapeutic intervention; and (c) analyses of the impact and benefit of occupation in daily life. 

*(1a) Use of an Occupational Perspective to Explain a Particular Phenomenon*. This category is based on a concrete situation, a specific conception of occupation, or a local reality. Occupation is used to explain a social behavior from the perspective of a previously defined theoretical construct. Authors that use this approach include Leive [26] and Cabello et al. [27].

In terms of conceptualizations and definitions of occupational science/occupation, Leive [26] focuses on the occupations of families. Occupations are evolving, flexible, orchestrated in parallel with other occupations, and purposeful. Therefore, behaviors that are involuntary, such as sleeping, are not occupations. However, going to sleep and waking up are occupations, as these acts possess a temporal-spatial structure, organization, and biological dimension.

To support this idea, Leive cites the following: Pierce's [28] concept of cooccupation, an occupation that requires synchrony within the occupational patterns of a cohabitating group; Zemke and Clark [14], emphasizing the crucial role of the environment in the construction of occupation, especially the family environment for children; Esdaile and Olson [29], noting that if occupational problems occur, the family dynamic is affected, including the orchestration of caregiver-child cooccupations and other familial roles; and Evans and Rodger [30], noting that the degree of synchronization between the caregiver and child impacts the construction of everyday occupations.

Occupations are performed as part of a routine that depends partly on others. An occupation has a name provided by society that reflects its meaning, is flexible in the face of daily requirements, is performed at a given time, and can be executed with mastery.

Occupations are made up of various tasks. Performing these tasks impacts growth and development. The conditions for existence underlying an occupation include the presence of cooccupations, such as a child going to sleep, which require a certain profile of person, such as a caregiver, to assist in performing them.

Leive asserts that it is necessary to develop certain behaviors before performing some occupations (e.g., going to sleep independently) and that synchrony between the caregiver and child constructs the child's occupation. Moreover, a suitable environment is required (such as the environment surrounding a child and caregiver). Finally, a routine is necessary to configure an occupation, which occurs through a progressive process.

For Cabello et al. [27], occupations are understood as part of everyday life and are performed habitually. Occupations are influenced by the occupations of others and modeled over time. As such, parents influence their children's occupations. The structure of an occupation impacts the family and children, driving behaviors. Occupational modeling consists of imitation (acting; doing) and imagination (preparing for action; thinking) and is influenced by interpersonal relationships. When there is disequilibrium, such as when a family member has a disability, occupations can become risk factors.

The authors cite concepts from Christiansen [31] to suggest that occupations impact social development and the emergence of habits and personal identity. Problems with participation in occupations are risk factors that affect the health of family members, especially children. The authors draw upon Nelson and Jepson-Thomas [32], noting that learning is an adaptive process that occurs through the performance of occupations.

Occupations and occupational knowledge impact a person's health. Occupations influence one's ability to adapt to society, develop habits, and identity and maintain relationships. Occupational modeling arises through interactions between people.

This perspective differs from those that follow because it does not (explicitly) take occupation as a therapeutic tool, but it allows accounting for different phenomena, a function that is also taken by authors in the following categories. 

*(1b) Elucidation of an Occupational or Occupational Science Concept to Support a Specific Therapeutic Intervention*. This group of authors proposes that a theoretical construct of occupation can be used to explain a behavior and perform a therapeutic intervention in a particular population. Authors using this approach include Drápela et al. [33]; Fernández et al. [34]; Imperatore [35]; Mella et al. [36]; Rodríguez and Gajardo [37]; and Torrico et al. [38].

In terms of definitions and conceptualizations of occupational science/occupation, Drápela et al. [33], in their investigation of persons deprived of their liberty, where they identified the meanings associated with criminal activity, indicate that occupation is human performance in response to the vital needs of an individual that allows one to meet social demands, express oneself, and develop an identity. Occupations provide a path towards meaning in life and the development of personal, cultural, and social identity. Occupations consist of meaningful activities; their effects include a sense of efficacy, and they are essential for achieving personal objectives and forging a strong self-concept. These elements are therefore developed through occupation in an occupational therapy intervention. In this way, in occupational therapy intervention, through the occupation, the previous elements are identified.

Fernández et al. [34] suggest that, in order for an occupation to be meaningful, it should support identity construction, provide a sense of belonging, and promote empowerment. This research is focused on productive enterprises of people suffering from mental health problems and shows how therapeutic intervention favors these enterprises and contributes to social rehabilitation.

Imperatore [35] reflects on occupation as the object of occupational science study in the context of sensory integration disorders, especially the impact of such disorders on occupation selection. The author notes that occupation consists of form, function, and meaning, based on concepts [14]. The author presents factors that influence occupation selection, with an emphasis on the “occupational metamorphosis” concept [39], which explains relationships among biological factors such as sensory-seeking behaviors. Thus, occupation functions as a therapeutic tool that affects a person's biology as well as their occupational performance. For this reason, therapeutic intervention by means of the occupation is a central axis in the treatment of sensory integration disorders.

The author cites Zemke [40], reflecting on the organization of occupation in space and time and its relationship with sensory processing. Time is described as the perception of change, including perception of simultaneity, subjective present, sequencing, and duration.

Mella et al. [36], in their investigation of persons deprived of liberty, focus on other considerations regarding occupation. For these authors, occupation (a) is a right and a fundamental element of society in everyday life; (b) has individual meaning and value provided by one's culture and context; (c) includes everyday tasks (self-care, leisure, and contributions to social and economic development of one's community); and (d) represents an intervention space for occupational therapy, as it serves as a potential rehabilitation tool consisting of an organized set of specific tasks and activities.

In the process of therapeutic intervention, the occupation guarantees the right to social integration, allows for emergence of a social order, and supports individual and social wellbeing (including for persons deprived of liberty). Its conditions for existence include everyday activities, which change according to the context and are structured as a function of a person's external conditions.

The authors assert that occupational science conceptualizations are relevant to occupational therapy practice. Theoretical occupational science foundations can be applied to interventions, including in the population of persons deprived of liberty. The authors suggest that these conceptualizations can be used to establish foundations for interventions. Outlining the stages of the intervention based on occupational science concepts ensures that the process will reflect a focus on occupational elements.

Rodríguez and Gajardo [37], in their research into the perceptions of elderly people about their use of public transport, note that occupation provides structure to life, drives habit development, and regulates affective relationships both with others and with the context. Occupation also offers a means to address psychological and behavioral symptoms. Therefore, its conditions for existence are the demands of the environment and the conditions of the person. The relevance of therapeutic intervention in this context is crucial to increase the quality of life of elderly people.

Torrico et al. [38] in their study on the occupational performance of a group of women note that occupations are affected by the rhythms of daily life and are expressed in both work and family contexts. The authors reflect on whether occupation arises from an individual or is determined by historical or cultural context. Occupation consists of form, function, and meaning.

In the intervention process, the occupation increases productivity; affects a person's life satisfaction; satisfies basic survival needs (self-care, shelter, safety, and sustenance); supports social structures (defense against predators and the environment); develops personal abilities; and affects health. Occupations may also generate or be influenced by risk factors including hardship, marginalization, alienation, and inequality [41], as well as gender prejudices that limit the occupations of women.

The principal condition for existence of occupation is the person, that is, the human as an occupational being in a sociocultural context. Occupational choices are determined by economic, political, social, and cultural conditions and are associated with social justice and equality.

To summarize, authors in this category consider occupation and occupational science in terms of therapeutic interventions with specific groups of users or patients. Like category (1a), this category uses occupational terminology to account for a problem, but it differs by proposing a specific therapeutic intervention. Thus, occupational science constructs are present in the intervention processes.

*(1c) Analysis of the Impact and Benefit of Occupation in Daily Life*. This category is used by a group of authors who address the benefits of occupation in general rather than for a particular population. These authors include Gómez [42]; Da Silva et al. [43]; Muñoz and Salgado [44]; and Rueda et al. [45].

In terms of the definition and conceptualization of occupation, Gómez [42] argues that occupation is a powerful agent capable of driving significant life changes. Occupations organize life, create a day-to-day identity, and provide existence with meaning.

Occupation has both a purpose and meaning. Purpose initiates the activity of occupation, and meaning is a complex, unconscious process mediated by affective and social elements.

Gómez notes that the study of occupation reveals the practical dimension of individuality that emerges through performing an occupation. That is, the concept of individuality is fundamental to the theoretical foundation of occupational science. The author emphasizes that the practice of individual occupations overlaps and intersects with other occupations, making it impossible to understand an occupation as an isolated entity. The intersections between occupations are evident in family, social, and work environments.

Da Silva et al. [43] understand occupation as a therapeutic strategy and central element in the development of occupational therapy. They note that the meaning of occupation changes according to the cultural context. For example, roles that are significant in occidental societies may have no meaning in nonoccidental societies. The authors point out that participating in occupations positively impacts a person's health.

In terms of the conceptualization and definition of occupational science/occupation, Muñoz and Salgado [44] propose that occupation includes a person's everyday tasks and may include activities performed during one's free time, such as relaxation, recreation, or leisure. Occupation has form, function, and meaning. In this approach to understanding occupation, the physical context is crucial and an object of occupational science investigation.

For Rueda et al. [45], occupation differentiates persons from other living beings and is constitutive of humanity. Participation in occupations can (a) generate an occupational equilibrium or disequilibrium; (b) emerge as the product of the time and place in which a person performs his or her activities; (c) provide an individual and collective sense to human groups; and (d) promote social belonging and strengthen the bonds among persons. In conclusion, occupations help to make sense of the project of life.

Authors in this category evaluate occupational context from various perspectives. They address the benefits of occupation in general. Thus, this category provides universal characteristics of the occupation applicable to every person. It does not focus on a specific population, as in category (1b), nor does it only explain a phenomenon such as in category (1a).

This approach, which views occupation and occupational science from a practical perspective or as a means to explain human behavior, differs from the next approach, which employs epistemological and theoretical elements to explain and analyze occupation and occupational science.

*(2) Occupation and Occupational Science as an Object of Study*. This approach is characterized by (1) reflection on occupational science/occupation concepts; (2) discussion of the approaches used to analyze these constructs; and (3) analysis of theoretical positions that impact practical occupational therapy knowledge and theory. These reflections are second-order analyses, associated with philosophy or epistemology.

This approach can be divided into the following categories: (a) reflections on theoretical occupational science/occupation constructs and (b) local theories on occupational science/occupation as an object of study.

*(2a) Reflections on Theoretical Constructs of Occupation or Occupational Science*. Authors in this category offer ways to understand occupational science/occupation from a reflective and epistemological perspective.

Morrison [46] offers a conceptual foundation based on philosophical methods which challenges occupational science to engage in a speculative and metareflective process. This philosophy of human occupation (Spanish acronym FOH, for* filosofía de la ocupación humana*) links rhetoric with science to analyze the development, structure, and dissemination of occupational science/occupational therapy knowledge, from an interpretive and critical position.

For Morrison, occupation is a phenomenon amenable to philosophical analysis, a component of clinical occupational therapy practice, a subject of occupational science investigation, and a political element. Morrison cites authors such as Wilcock [41], emphasizing her theory of the occupational nature of human beings, developed through reflections on policies, ecosystems, and relationships between the person and the environment; Kronenberg et al. [47], emphasizing their political-social perspective; and Carrasco and Olivares [2], noting that the construct of occupation implies interdependence between the subject and the constructed reality.

Morrison et al. [48] also conceptualize FOH as a discipline that studies the structure of occupational therapy/occupational science knowledge, developing metatheories that allude first to scientific knowledge and then to scientific practice. The authors support their propositions by citing Díez and Moulines [49], Echeverría [50], Longino [51], and Marcos [52].

FOH addresses knowledge, learning, and internalization of information. The process allows for a critical approach towards the acquisition of occupational therapy knowledge and its translation into actions within the context of professional practice.

Paradigms, therefore, operate within both theory and practice. Paradigms facilitate articulation of key occupational therapy/occupational science concepts and illustrate the evolution of scientific knowledge within the social context of the professionals who generate the findings.

The article also applies Morín's complexity paradigm [53] to the study of occupational therapy/occupation. The advantage of this approach is its divergence from linear, mechanistic paradigms [54]. The notion of complexity liberates the authors from the health-sickness dichotomy and accommodates the totality of factors affecting quality of life. Conceptualizations derived from the complexity paradigm can be applied to occupational science, including multidimensionality and the impulse towards multidimensionality and critical thinking that leads to the problematization of previously taken-for-granted concepts.

Finally, the authors propose a social paradigm of occupation, based on Kuhn [19]. This paradigm considers the evolution of scientific knowledge within the social context of the professionals who generate the findings. The paradigm remains in constant development, positioning occupation as a complex, systematic concept with economic, political, health-related, cultural, and social dimensions that affect the equity and wellbeing of communities.

Ramírez and Schliebener [55] propose the use of dialectics to generate occupational science research and analysis methods. These authors examine occupation in relation to existing narratives, including the occupational reflections of characters found in classical Chilean literature.

The authors consider occupation to be a complex phenomenon that undergoes constant transformation, as its medium (the environment and the person) also experiences continual change. Occupations are understood in the local context and performed by people in the here and now, but each occupation also has a history, essence, and distinctive character that differentiate it from other occupations. The concept, therefore, possesses both an objective dimension, based on reality, and a subjective dimension, based on individual learning. The occupational action executed by a person represents the synthesis of both factors. This action consists of individual elements, which have distinctive features, and common elements, which remain constant in different places and times.

Ramírez and Schliebener posit that occupations create and transform society, changing recursively in the face of emerging requirements. Occupations also influence personal identity, thereby changing the way that a person performs other occupations. Occupations are based on material and historical conditions but are also influenced by the ideas of the performer. That is, occupations are performed in given a space and time in a way that involves both objective (social) and subjective (individual) elements. Certain conditions are required for each occupation, which are determined by other, previously performed occupations, implying a historical development of occupation.

The authors also argue for the utility of historical materialism to justify interventions in a specific population (historical materialism is an epistemological explanation that accounts for how human history has developed. Based on the ideas of Karl Marx and Friedrich Engels, historical materialism positions material conditions of existence as a basis for understanding juridical relationships and forms of state and of all civil society [56]. Thus, to think of human occupation from this perspective implies understanding the material historicity of occupations and how this explains, for example, occupational performance). These authors use dialectics as a foundation to study occupation, noting that its complexity makes it useful for elucidating this multifaceted concept. The authors view dialectics as the science of the general laws governing nature, avoiding the partialities associated with the subject performing the occupation. The authors consider a materialist perspective to be essential for comprehending occupation and its inherently subjective elements from an objective standpoint [57].

Rueda [58] redefines the concept of occupation based on hermeneutic and phenomenological rules [59]. Hermeneutics is used to generate a new reading of occupation by examining previous texts from various authors. Phenomenology is used to understand occupation as a pivotal point of human experience as well as explore the significance of this experience.

The author interprets occupation as a concept that involves a speculative knowledge or an attempt to comprehend the world, including the facet of the world called occupation.

Based on historical texts, the author notes that occupational therapy brought together biomedical and humanist knowledge from different perspectives. These views included content from studies conducted outside of Chile, but applicable to the Chilean context through hermeneutics.

Rueda also considers occupation from a bioethical. The author underscores concepts essential to occupation as understood in occupational therapy: knowing, doing, formal education, professional practice, models, techniques, and praxis. These concepts are based on Lolas [60], who notes that some characteristics of professional practice are defined by society. Professional practice includes the translation of knowledge into action; moreover, the author distinguishes between those who create and those who apply knowledge. The latter group ultimately fulfills the goals of professional practice. Specific professional actions are expressed through models, which facilitate deeds well done that articulate the knowledge, techniques, and interests of a profession.

Rueda notes that, in generating a body of knowledge and validating scientific findings, it is important to remain conscious of the discipline's theoretical framework. Therefore, if occupational science is incorporated into occupational therapy, the development and evolution of the two fields go hand-in-hand.

Authors in this category use different theoretical and philosophical approaches to reflect on occupation and occupational science but do not propose a redefinition of concepts.

*(2b) Local Theories on Occupation or Occupational Science as an Object of Study*. This second category applies the study of occupational science/occupation to local realities and, unlike the previous category, proposes a redefinition of concepts. The authors that use this approach are Álvarez et al. [61], Carrasco and Olivares [2], and Da Silva and de Araújo [62].

Álvarez et al. (2010) propose a theoretical construction of occupation, with conceptual bases including (a) Latin American perspectives on knowledge and (b) the epistemology of historical consciousness, citing Zemelman in both instances [63, 64]. The first element suggests that Latin American concepts have developed out of investigations into the social world, incorporating components such as subjects, collective subjectivities, and social movements, and an awareness of history and politics. The second element focuses on the ethics and politics of generating knowledge, opening new, coherent horizons that contrast historically fragmented perspectives on knowledge.

The authors note that the reconceptualization of the term occupation provides opportunities for discussion and growth within the discipline. Recognizing that national conceptualizations are based largely on international works, they applied the international literature to the Latin American process, emphasizing the need to define occupational science in the national context. The authors propose a local definition that is the product of long-term reflection: occupation is a meaningful everyday activity, performed by a person and named by the culture.

Carrasco and Olivares [2] cite Kuhn's paradigm [19] to support the assertion that occupational science/occupational therapy professionals hold the concept of occupation central to their disciplines despite different nuances in interpretation. The authors propose that general systems theory (GST) [65, 66] supports a deep understanding of occupation, as it conceptualizes human beings as high-complexity systems and therefore drives articulation of social and natural scientific findings through multidisciplinary studies. They further suggest that dynamic systems theory (DST) [65, 67, 68] may facilitate a more comprehensive understanding of occupation. DST goes beyond the hierarchical relationships established by GTS to encompass heterarchical associations. Furthermore, DST captures the dynamic nature of complex systems. By citing the theoretical constructs used in the field, Carrasco and Olivares apply a constructivist approach [69], emphasizing the role of occupational therapists in developing the theoretical foundation of their discipline.

The authors approach occupation as an element that can be understood through human and social dimensions and as the object of occupational therapy investigations. Occupation is a phenomenon that can be studied and explained, as it is performed and observed in a given environment.

Carrasco and Olivares refer to authors who approach occupation as an object of occupational science investigations that consists of observable elements, such as form, as well as interpretable elements, such as meaning and purpose. The study of occupation promotes development of the academic discipline and clinical occupational therapy practice.

The authors also note that occupation is not synonymous with activity [28] as perceived by individuals or society in general. Occupation is a discrete act and experience perceived as distinct by the person who performs it. This experience is individual and singular. Occupations are different each time they are performed due to the interaction among the various components, evolving across the lifespan.

The authors reference Kielhofner [70] to illustrate that occupational therapy has a unique perspective shared across the discipline, despite nuances that surface in applications to different contexts. The authors also cite other sources that define occupation: (a) Nelson [71], who proposes that the significance of occupation lies in its symbolic meaning, which emerges in a given space and time, and is therefore singular, and (b) Iwama [72], who emphasizes the individual meanings of occupation that materialize in different cultural and social contexts.

Carrasco and Olivares conceptualize occupation as a result of dynamic interactions between activity, purpose, occupational form, and meaning.

In sum, the authors propose a concept of occupation based on three types of constructions: (a) the habitual and historic occupational therapy factors—that is, person, environment, and activity—which are not subject to direct observation or analysis, as it is impossible to separate the person from their environment or activity; (b) factors that can be perceived and analyzed by an observer, that is, the occupation's form, function, and meaning; and (c) factors only interpretable by the person performing the occupation.

Occupation also has certain conditions for existence, such as association with the human and social spheres. These factors are evident when occupation is approached from a subjective perspective. This means that there are many ways to understand occupation that depend on the observer's viewpoint and context and therefore many ways to explain reality. Categories, which emerge through interactions among people (intersubjectivity) in a given situation (context), can facilitate such understanding. These concepts explain “reality” but they are not reality.

The study by Da Silva and de Araújo [62] reflects on local contextualization of occupational science/occupation elements in the practice of occupational therapists working in the Amazon rainforest of Pará, Brazil. These authors cite Clark and Lawlor [73], noting that occupational science is an emerging discipline that understands human beings as occupational beings. The authors emphasize the associations between commitment in the context of occupation and the context of human life, especially the influence of this relationship on health, wellbeing, and social participation, citing Rudman et al. [74] to demonstrate the role of occupation in supporting health and participation.

These authors, therefore, view occupation as participation in everyday activities: self-care, leisure, work, and so forth. The definition of occupation is ambiguous within the discipline, and the term activity is sometimes used interchangeably with occupation. Occupation encompasses a diversity of actions carried out by people in a given context. It is a type of activity that occupies a person within a social context and integrates human expression.

Moreover, occupation includes the activities carried out by a person (who may vary according to age, race, sex, etc.) in the context of work, leisure, and social activities during one's everyday life (which in turn encompasses many contexts influenced by one's culture). Occupations are essential for a person's life, health, and wellbeing; they allow a person to organize his or her world; they are dynamic; and they evolve throughout the lifespan.

Occupation also has therapeutic value that transcends the everyday. Occupation in this sense can generate change, as it touches the social, cultural, economic, biological, and philosophical spheres. The authors approach occupation as a broad subject, noting that a clear description of occupation supports effective occupational therapy interventions.

Occupations consist of actions and meanings that evolve over a person's lifetime, generating change and transformation. Therefore, occupations have a meaning in a given context, provide structure, and endow a person with humanity; their social context is the condition for their existence.

Authors in this category, although employing a series of theoretical constructs that come from different fields of knowledge (especially in the United States), manage to reconstruct or contextualize the occupation for relevant perspectives with the local reality, which enriches regional theoretical and reflexive development.

## 4. Discussion and Conclusion

This study identified the main approaches to understanding and applying concepts of occupation and occupational science in the literature of the discipline as published in the RChTO between 2001 and 2012. These findings document the diversity of the discipline and contribute to the education of new occupational therapists.

This analysis made it clear that many positions can be used to study occupation. Approaches to understanding occupational science are informed by concepts of occupation, based on literature relevant to authors in occupational science and related fields. The analysis clearly revealed two different approaches. The first considers occupational science/occupation from a practical perspective and as a means to explain human behavior; the second considers occupational science/occupation as an object of study. These approaches were developed on a regional level and have been used as a foundation for local professional practice.

The first main approach is characterized by the application of occupational science/occupation constructions. This approach includes three categories: the use of an occupational perspective to explain particular phenomena; application of occupational science/occupation constructions to therapeutic interventions; and the study of the impact and benefits of occupational science/occupation in daily life.

The second main approach focused on occupational science/occupation as an object of study and can be divided into two branches. One is based on the global analysis of theoretical constructs while the other offers local perspectives on occupational science and occupation.

The latter refers rather to a reconstruction, contextualization, or adaptation of different international theoretical perspectives, and not to the creation of other theories to explain occupation. Thus, the value of this category is that it finds ways to apply international theories at a local level, making them culturally relevant.

A noteworthy finding of this study is that there were only two Latin American theoretical proposals offered in RChTO publications (2001–2012) [2, 55]. Likewise, only one original Latin American definition appeared [61].

In light of the above, we asked about the characteristics of the diffusion of knowledge of occupational therapy in Chile. In Chile and in neighboring countries, the theoretical contributions are generated by groups of academics from the main state universities. In the earlier publications, between the years 1960 and 1980 approximately, a reductionist biomedical paradigm is perceived, which has marked the pattern of practice of the profession in the region. However, the concern to include social participation spaces is not neglected. Later publications are concerned with the empowerment of individuals and groups in their own processes of health maintenance and recovery. In these later publications, the focus is not on the disease but on the person in his or her own context, and on the opportunities that context offers for activities that can be understood as healthy in terms of their meaning for the individual, linked to his or her history, beliefs, and life within a cultural environment.

These results lead to the following questions: What barriers to developing local knowledge do Latin American occupational therapists face? Do Latin American occupational therapists have the motivation, time, and training necessary to carry out research and publish manuscripts? Is the habit of reflecting on interventions an established part of professional practice? Does the discipline, on a Latin American level, place importance on the systematization of professional practices and development of evidence? On an institutional level, are there incentives and support to develop research competency and dissemination of knowledge?

Addressing these questions is crucial to supporting and promoting occupational science/occupational therapy research, a strong professional identity, evidence-based practice, continuing education, scientific societies, and application of research to professional practice. Academic staff responsible for developing occupational therapy curricula should review the competencies needed to improve the discipline and integrate research as a dimension of professional practice.

This study illustrates the recent directions of the discipline at the regional level, examining the written expressions in a particular Spanish-language occupational therapy journal. Therefore, the findings are applicable only to this specific geographic and cultural sphere. It would be interesting to ask the following question: What other fields and media develop and publish reflections and studies of occupational science/occupation?

It seems appropriate to investigate whether studies such as the present have been conducted in other Latin American countries since there are cultural similarities between them, due to their common history. In this way, it would contribute to a broader and deeper knowledge, possibly applicable to the reality of this region, in the areas of theory and practical knowledge. It is also clear, in our view, that the development of knowledge should consider the exercise of distinguishing local knowledge from universal disciplinary knowledge.

Thus, this study provides a new perspective on the way that authors in the field apply occupational science/occupation concepts. It is possible that other sources of literature would yield different approaches for understanding occupational science/occupation concepts.

This study focused on the historical context and temporal elements of the descriptions of occupation in each article. Research to address occupation from cross-sectional (social-historical-cultural) and longitudinal perspectives (current views, issues relevant to the “here and now,” capturing unique and unrepeatable moments in time) would also be useful.

This article contributes to the systematization of data relevant to professional practice and provides insights on fundamental concepts such as occupation. The article illustrates current trends in the discipline in terms of research, practice, and education of occupational therapists.

New conceptualizations offered by Latin American authors enrich international development of occupational science and, therefore, the occupational therapy profession.
